# Development and Validation of a Questionnaire on Breastfeeding Intentions, Attitudes and Knowledge of a Sample of Croatian Secondary-School Students

**DOI:** 10.3390/children5050056

**Published:** 2018-04-27

**Authors:** Marija Čatipović, Martina Marković, Josip Grgurić

**Affiliations:** 1 General Pediatric Office Marija Čatipović, 43000 Bjelovar, Croatia; 2 Association of the parents of children with special needs “Bjelovarski leptirići”, 43000 Bjelovar, Croatia; martina.markovic91@gmail.com; 3 UNICEF Office for Croatia, 10000 Zagreb, Croatia; jgrguric@unicef.hr

**Keywords:** questionnaire, breastfeeding, students

## Abstract

Background: Validating a questionnaire/instrument before proceeding to the field for data collection is important. Methods: An 18-item breastfeeding intention, 39-item attitude and 44-item knowledge questionnaire was validated in a Croatian sample of secondary-school students (*N* = 277). Results: For the intentions, principal component analysis (PCA) yielded a four-factor solution with 8 items explaining 68.3% of the total variance. Cronbach’s alpha (0.71) indicated satisfactory internal consistency. For the attitudes, PCA showed a seven-factor structure with 33 items explaining 58.41% of total variance. Cronbach’s alpha (0.87) indicated good internal consistency. There were 13 knowledge questions that were retained after item analysis, showing good internal consistency (KR20 = 0.83). In terms of criterion validity, the questionnaire differentiated between students who received breastfeeding education compared to students who were not educated in breastfeeding. Correlations between intentions and attitudes (r = 0.49), intentions and knowledge (r = 0.29), and attitudes and knowledge (r = 0.38) confirmed concurrent validity. Conclusions: The final instrument is reliable and valid for data collection on breastfeeding. Therefore, the instrument is recommended for evaluation of breastfeeding education programs aimed at upper-grade elementary and secondary school students.

## 1. Introduction

The World Health Organization (WHO) and United Nations Children’s Fund (UNICEF) recommend initiation of breastfeeding within an hour of birth, exclusive breastfeeding for the first 6 months of life, and continued breastfeeding beyond 6 months and at least up to 2 years of age or more along with the introduction of nutritionally adequate and safe complementary foods [1]. Optimal breastfeeding is so critical that it could save the lives of over 820,000 children under the age of 5 years every year [2]. However, not enough mothers breastfeed their infant within the first hour of the infant’s life [3,4], only about 36% of infants aged 0–6 months worldwide were exclusively breastfed over the period of 2007–2014, and in many countries, less than a fourth of infants 6–23 months of age meet the criteria of dietary diversity and feeding frequency that are appropriate for their age [2].

To improve the described situation, there are many support and promotional breastfeeding activities undertaken in Croatia: breastfeeding support groups [5], pregnancy courses [6], children’s counseling centers-friend of breastfeeding [7], “Nursery-children’s friend” programs [8], “10 Steps to protect, support and promote community breastfeeding” [9], “County friend of breastfeeding” [10], “A kindergarten friend of breastfeeding” [11], and the destigmatization of breastfeeding in public and other breastfeeding promotion activities. Due to significant negative effects of formula marketing [12] we have put a special effort in working with pharmaceuticals [13] and the public promotion of breastfeeding [14]. However, results still remain unsatisfactory [15].

We think that one of the important reasons why the results are not satisfactory is because the breastfeeding promotion and education activities are mostly directed at adults. Adolescents are more successful at retaining information and having positive attitudes about breastfeeding [16,17]. The development and implementation of breastfeeding education programs in school is a vital component of breastfeeding promotion initiatives [18]. It is very important to focus the activities not only on girls but also on boys [19]. For that reason, in the past 10 years, researchers have focused on promotional and educational activities to work with children and the youth [20,21].

Implementation of breastfeeding education programs in school settings is not governed by competent authorities [22]. Therefore, the analysis of adolescents’ level of knowledge and intentions to breastfeed and examination of the effect that a structured breastfeeding education program might have on the potential change in their knowledge, attitudes and intentions are the first steps in the process of showing that breastfeeding education programs should be implemented in school settings. [23]. However, researchers have faced the problem of not having a validated tool to perform such analyses.

The main objective of this paper is to construct an objective, reliable and valid instrument that would assess adolescents’ attitudes, intentions and knowledge about breastfeeding, which would be used in our region regarding our social, cultural, economic, historical, health, legal and other specificities.

## 2. Materials and Methods

### 2.1. Ethical Consideration

Ethical approval for conducting the research was given by the Ministry of Education, Sport and Science. Approval for conducting research at high school Bjelovar was given by the school’s Ethical committee. Students were allowed to participate in the study only after they read an informed consent and gave agreement that they were willing to participate in the study, according to Croatian law [24].

### 2.2. Procedure

Prior to a study on validity and reliability of the questionnaire, the authors conducted a pilot study with 30 medical secondary school students. The aim of that study was to assure that all students understood the questions and that those questions were neither ambiguous nor suggestive. The authors considered all of the students’ objections and suggestions and accordingly modified the items.

A study with original and comparison groups was conducted on-line. Students were given an on-line Breastfeeding intentions, attitudes and knowledge questionnaire (hereafter: BIAKQ).

The survey was conducted on-line on the school’s computers, thus avoiding the possibility of revealing participant’s identity via home computer’s IP address. Data collection was carried out on the same day for all students in order to minimize communication about questionnaire among students which could have an impact on results of the study. Before students started to fill in the questionnaire, the principal author introduced them to the purpose, eventual risks and benefits of the study, and with safety measures (for the data and for students themselves) [25,26]. Students were also informed about constructs that could have confused them: exclusive breastfeeding, milk formula, public breastfeeding, emotional attachment, etc.

### 2.3. Informations for Students Before and After the Research

It was important that all students were given the information that correct answers exist only for knowledge questions. As for intentions and attitudes statements, they were informed that they should choose the answer which corresponds the most with their current motives and choices. Students were also explained that for male students, items of intentions are formed in terms of giving support to their partner and for female students, items of intentions were formed in terms of breastfeeding or not breastfeeding.

Students were also given the study director’s contact information and invitation for voluntary participation in breastfeeding promotion activities within the association “For healthy and happy childhood”. While filling in the questionnaire, students were monitored by the school professors, meaning that the authors of the study were not present in classrooms.

After the research, the principal author organized the education where all of the knowledge questions were answered and guidance for advisable intentions and attitudes was given.

### 2.4. Questionnaire

The IIFAS [27] questionnaire was only the outset for questionnaire construction, since it is aimed at grown-up women who already have children. Therefore, the authors had to construct more questions that would be appropriate for targeting the population: secondary school students. The first version of the questionnaire consisted of 8 breastfeeding intentions and 15 knowledge items. After the literature overview and preliminary research, the authors concluded that the questionnaire should be extended. Therefore, the authors generated 10 more intentions items, 29 more knowledge items and 39 items about attitudes toward breastfeeding.

The first part of the questionnaire included information about socio-demographic characteristics and information regarding breastfeeding exposure (being breastfed as a baby, longevity, reasons for discontinuation, etc.).

The second part encompassed items about the intention to breastfeed. Students were given 18 items that refer to the intention to breastfeed. Students evaluated their intentions to breastfeed/support their partner to breastfeed on a Likert scale by giving a grade of 1 (strongly disagree), 2 (disagree), 3 (neither disagree nor agree), 4 (agree) or 5 (strongly agree).

Items regarding attitudes toward breastfeeding constituted a third part of a questionnaire. There were 39 items that referred to attitudes towards breastfeeding. Students again evaluated their attitudes toward breastfeeding on a Likert scale.

Finally, the fourth part of the questionnaire consisted of 44 various breastfeeding knowledge questions. Possible answers were “true” or “false”.

### 2.5. Sample Description

Inclusion criteria included attending the third and fourth grade of high school, coming to testing on time, giving informed consent and a duly completed questionnaire. Students who already had children were excluded from the research.

The study examined three samples. The first sample included 112 third-grade (16.71 ± 0.576 years of age, 60.7% female, 39.3% male) high school students from Bjelovar (Croatia) who did not participate in any breastfeeding education module. The second group of students was 111 fourth-grade high school students (17.73 ± 0.466 years of age, 59.5% female, 40.5% male) who completed a breastfeeding education module. Finally, there were 54 fourth-grade medical secondary school students (17.24 ± 0.473 years of age, 75.9% female, 24.1% male) who did not complete any breastfeeding education module but they often encountered breastfeeding through their theoretical and practical education. In Croatia, medical secondary school students differ from high school students by having 5 instead of 4 years of secondary school and by having different curricula which prepare them to be health workers (nurses, physiotherapist, dental technicians, laboratory technicians, etc.), while high school prepares one to go to college.

### 2.6. Validity Assessment

Third-grade high school students who were not educated about breastfeeding were examined to test the characteristics of the BIAKQ and presented an original sample.

Fourth-grade high school students who were educated about breastfeeding served as a comparison group to the third-grade high school students when comparing their questionnaire results. They were not used to test the characteristics of the questionnaire (e.g., for item analysis, factor analysis and reliability assessment).

Fourth-grade medical secondary school students served as a comparison group to high school students who underwent breastfeeding education. They were chosen by design, due to the fact that they often meet with breastfeeding matter through other subjects in school, within the curriculum for health-care workers. It will be noted that breastfeeding as a separate topic is not included in the secondary-school curriculum of medical secondary school either.

The validity was assessed through comparison between students who underwent a special breastfeeding education module and students who were not specifically educated in breastfeeding.

### 2.7. Statistics

Statistical indicators used in the procedure regarding keeping or excluding items from the final form of the questionnaire were: inter-item correlations (seen from the correlation matrix), arithmetic mean (M), standard deviation (SD), corrected item total correlation (Itc), and squared multiple correlation (*R^2^*). Explanatory factor analysis with principal components extraction method has been carried out in order to reduce the number of interpretable components. Factor analysis is a technique that is used for identifying groups or clusters of variables. This technique has three main uses: to understand the structure of a set of variables, to construct a questionnaire to measure an underlying variable and to reduce a data set to a more manageable size while retaining as much of the original information as possible. The authors have used the Guttman-Keiser criterion and Scree plot diagram to retain variables that are most informative among variables. Components which satisfy all these criteria and explain more that 55% of the variance have been retained. To assess reliability, the Cronbach α coefficient was used for intentions and attitudes, and KR-20 for the knowledge test. Procedures used for validity examination were independent *t*-tests, the Mann-Whitney U-test and Pearson’s correlation coefficient.

## 3. Results

Sociodemographic data are shown in Table 1.

## 4. Breastfeeding Intention Scale Analysis

### 4.1. Breastfeeding Intentions Items and Factor Analysis

Initially, 18 items were included in the breastfeeding intention scale. After the primary item analysis, which included inter-item correlations, M, SD, Itc and *R^2^*, only 10 items have been retained and 8 items have been excluded.

Prior to explanatory factor analysis, the authors examined Barlett’s test of sphericity and the Keiser-Meyer-Olkin Measure of sampling adequacy (KMO). They indicated that correlation matrix was suitable for running the factor analysis. Promax rotation was selected in order to interpret the results more easily. Table 2 shows the way items were loaded on 4 components, and Table 3 shows the factor analysis results. Components were named as follows: “Breastfeeding and returning to work”, “Breastfeeding when/as long as mother and/or child desire” and “Breastfeeding in public” (this component encompassed 2 items). Intentions to breastfeed in public could be properly examined with a small number of questions (sometimes only one question could be enough to see whether one is ready or not to breastfeed in public), so this component was retained despite that it includes only two items. Also, there were two items that saturated the fourth component. These items describe a negative intention towards breastfeeding, i.e., a positive intention to formula feeding. Therefore, the component was called “Lack of interest in breastfeeding”. The total score for the intention scale should be computed as the sum of all items.

### 4.2. Reliability Assessment of Breastfeeding Intentions Scale

Table 4 shows inter-item statistics and reliability of the scale.

Correlation coefficients range from 0.214 to 0.569 and indicate moderate correlation. Half of the items have correlation coefficient above 0.4, which is considered satisfactory.

Reliability in terms of internal consistency was examined. Cronbach’s alpha coefficient was 0.713. The value of the coefficient depends on the number of items and their inter-correlations. Commonly accepted standards account for Cronbach’s alpha coefficient above 0.90 for very reliable, above 0.80 for highly reliable and above 0.70 for satisfactory reliable measures.

### 4.3. Validity Assessment Results for Intentions

The Kolmogorov-Smirnov test showed that distribution is normal (d = 0.057, *p* > 0.05); therefore, the difference was examined using an independent *t*-test. Comparisons are shown in Table 5. High school students who received breastfeeding education have more positive intentions toward breastfeeding (M = 35.24, SD = 5.82) compared to students who did not receive this kind of education (M = 32.79, SD = 5.901), t (221) = 3.132, *p* < 0.005. High school students who were educated about breastfeeding had more positive intentions (M = 28.6, SD = 4.11) than medical secondary school students who were not educated but who are exposed to the breastfeeding topic in class (M = 25.18, SD = 2.65), t (163) = 2.39, *p* < 0.05. 

Students who want to learn more about breastfeeding at school have more positive intentions toward breastfeeding (M = 35.32, SD = 6.235), t (221) = −3.420, *p* < 0.05 compared to students who do not want to learn more about breastfeeding at school (M = 32.64, SD = 5.386).

Female students who were not breasted or who were breastfed up to 3 months of age have less positive intentions towards breastfeeding (M = 30.00 SD = 6.759) compared to students who were breastfed for more than 3 months (M = 34.66, SD = 5.286), t (66) = −3.175, *p* < 0.05.

## 5. Breastfeeding Attitude Scale Analysis

### 5.1. Breastfeeding Attitude Items and Factor Analysis

After the item analysis was performed, 33 out of 39 items were retained for further analysis and 6 were excluded.

Barlett’s test of Sphericity and KMO were tested prior to running the factor analysis, and they showed that a correlation matrix was suitable for running the factor analysis.

According to Keiser’s criterion, there were 9 possible models that yielded components with Eigenvalues higher than 1. According to the proportion of total explained variance, there were only three models that explained more than 55% of the variance and that were acceptable for further analysis [28]. These were models with 7, 8 and 9 extracted components.

**Figure 1:**
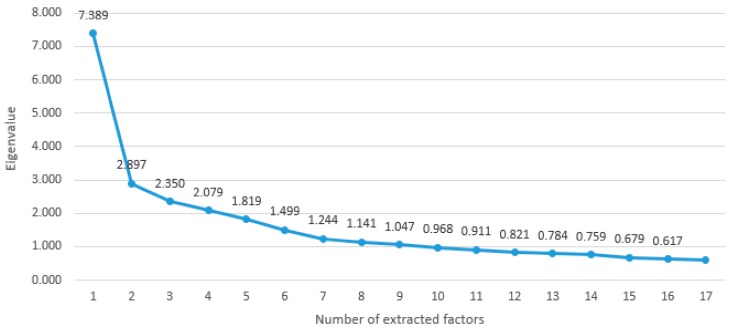
Scree plot test-ratio of the number of extracted factors to the Eigenvalues.

Considering the Scree plot test (Figure 1), the optimal number of extracted components is seven. With 7 components for Keiser’s criterion (Eigenvalue 1.184) being satisfied, the proportion of cumulative explained variance is 58.413%. Varimax rotation was selected in order to interpret results more easily. Items loaded on 7 components as follows:

Table 6 shows which items loaded on which component. There are 7 components: “Breastfeeding in public” (Cronbach α coefficient 0.843), “Father’s role in breastfeeding” (Cronnbach α coefficient 0.772), “Breastfeeding on the day of delivery” (Cronbach α coefficient of 0.820), “Breastfeeding at the workplace” (Cronbach α coefficient 0.716), “The benefits of breast milk over formula milk”. (Cronbach α coefficient was 0.693), “Prejudice-based attitudes towards breastfeeding” (Cronbach’s α was 0.576) et “Longevity of breastfeeding” (Cronbach’s α of 0.70). Five out of seven factors have an Cronbach alfa coefficient 0.70 or higher, which means that they are highly internal consistent. The lowest saturation coefficient was 0.422 is high enough and supports the reliability of the corresponding factors.

### 5.2. Reliability Assessment of Each Factor of the Breastfeeding Attitude Scale

The authors tested the arithmetic mean (M), variance (V), Corrected item-total correlation and Cronbach’s alpha value if deleted items.

The corrected Item-Total correlations range from 0.252–0.766. Cronbach α of the entire scale is 0.871. Factor five contains only three items and its reliability coefficient is marginal (α = 0.689). As Cronbach α directly depends on number of items per scale, it is certain that this value would increase by adding one or two more items. As for factor 6, its reliability coefficient is far below 0.70 (α = 0.599), which means it is heterogeneous and should be interpreted with caution. Although Factors 5 and 6 do not reach a satisfactory internal reliability value of 0.70 they were retained in the scale because the reliability of the entire scale was good.

### 5.3. Criterion-Related, Known-Groups and Concurrent Validity Results for Attitudes

The Kolmogorov-Smirnov test showed that the distribution is normal (d = 0.050, *p* > 0.05); therefore, the difference was examined using an independent *t*-test. High school students who received breastfeeding education (*n* = 111) had more positive attitudes towards breastfeeding (M = 129.74, SD = 14.030) compared to students who did not receive this kind of education (M = 122.31, SD = 13.957, *n* = 112), t (221) = −3.963, *p* < 0.001. Students who want to learn more about breastfeeding at school (*n* = 114) have more positive attitudes towards breastfeeding (M = 131.77, SD = 13.374), t (221) = −6.659, *p* < 0.01 compared to students who do not want to learn more about breastfeeding at school (M = 119.98, SD = 13.049 *n* = 109). There is a positive correlation between the desire to learn more about breastfeeding at school and place of residence (r = 0.203, *p* < 0.05). Students from larger cities are less likely to have a desire to learn more about breastfeeding at school when compared to students from rural areas.

Female students who were not breastfed or who were breastfed up to 3 months of age (*n* = 33) have less positive attitudes towards breastfeeding (120.82, SD = 14.063) compared to students who were breastfed for more than 3 months (*n* = 35, M = 129.71 SD = 12.872), t (66) = −2.723, *p* < 0.05. Comparison is shown in Table 7.

Pearson’s correlation coefficient revealed a moderate correlation: r = 0.494, *p* < 0.01, indicating that students who have more positive attitudes towards breastfeeding also have more positive intentions to breastfeed.

## 6. Breastfeeding Knowledge Item Analysis

### 6.1. Breastfeeding Knowledge Items

Inter-item correlations range from −0.197 to 0.530. There were many items that correlated negatively or non-significantly with other items and for that reason have been excluded. There were 31 items that have been excluded, and 13 items that have been retained. The final result is calculated as the sum of all correct answers and it represent one’s knowledge about breastfeeding.

### 6.2. Reliability and Validity Assessment Results for Knowledge Questions

Internal consistency was examined with the Kuder–Richardson 20 (KR-20) formula, since all items were codified as dichotomous (correct/incorrect). The KR-20 for 13 items was 0.831.

The Kolmogorov-Smirnov test showed that the distribution is asymmetric (d = 0.311, *p* < 0.01); therefore, the difference was examined using the Mann-Whitney U-test. High school students who had been educated about breastfeeding (*n* = 111) had better knowledge about breastfeeding (Mean rank = 128.80) compared to high school students that had not been educated (*n* = 112, Mean rank = 94.26), U = 8.203, *p* < 0.01.

The Mann-Whitney U-test between high school and medical secondary school students revealed a significant difference between the two groups regarding the knowledge test score six months after education: U = 1.4285 *p* < 0.01. High school students showed better knowledge (Mean rank = 90.86) than medical secondary students (Mean rank = 53.95). It will also be noted that before education, students did not statistically differ regarding knowledge: U = 2.5975 *p* > 0.05; medical secondary school students had the same breastfeeding knowledge (Mean rank = 75.60) as high school students (Mean rank = 79.28).

Pearson’s correlation coefficient revealed a moderately significant correlation between the knowledge test and attitude scale: r = 0.379, *p* < 0.01. The correlation with intentions was also significant, r = 0.290, *p* < 0.01.

## 7. Discussion

We know that attitudes toward breastfeeding are already formed in preadolescence, so it is logical that the focus of promotional and educational activities on breastfeeding is directed at the young [29,30]. Schools are a perfect place to carry out the activities of raising awareness of breastfeeding and breastfeeding education of young people [31].

However, Croatia has not provided breastfeeding education in elementary and secondary education curricula [32]. Schools need to be offered an effective breastfeeding educational program which is not possible without evaluation of the program [21,23,33]. It is not possible to compare activities that are not methodologically uniform and measurable. We have defined by the literature and in consultation with our colleagues, the initial educational intervention [33], that we plan to develop into a program, but for the evaluation of the program, we needed an appropriate questionnaire. 

An attempt was made to use the questionnaires that already exist. The IFFAS questionnaire is intended for women who have given birth [27], as well as a questionnaire used in the “Baby-friendly Hospitals Initiative” [34], followed by the Irish National Infant Feed Survey [35] and a variant of the Nigerian questionnaire [36], and other similar questionnaires [37]. Questionnaires are also available for pregnant women [38], midwives [39] and nurses [40]. Some breastfeeding questionnaires targeted at young people are focused on specific situations, such as breastfeeding at work [41], some are available against payment, others only examine knowledge and attitudes but not intentions, some are not validated, etc.

For that reason, the authors started to develop their own questionnaire that would be used in their area. The validity of the questionnaire in this research was confirmed in several ways. After analyzing items, conducting factor analysis and assessing reliability, the authors decided to examine whether the scale differentiates between intervention groups, e.g., was the scale valid in terms of criterion-related validity? If the scale is valid, one could expect to find more positive intentions, attitudes and knowledge toward breastfeeding among students who were educated about breastfeeding in contrast to students who did not participate in a breastfeeding education program. As expected, students who received breastfeeding education had better knowledge (Mean rank = 128.80) and more positive intentions (M = 35.24, SD = 5.901) and attitudes toward breastfeeding (M = 129.74, SD = 14.030) compared to students who did not receive this kind of education (knowledge: Mean rank: 94.26/intentions: M = 32.79, SD = 5.82/attitudes: M = 122.31, SD = 13.957). This result indicates that the breastfeeding intentions, attitudes and knowledge scales are valid in terms of evaluation of some education program regarding breastfeeding.

Furthermore, some previous research has shown that students who want to learn more about breastfeeding at school have more positive intentions and knowledge about breastfeeding [33]. Results show that students who want to learn more about breastfeeding at school have more positive intentions (M = 35.32, SD = 6.235) and attitudes (M = 131.77, SD = 13.374) toward breastfeeding compared to students who do not want to learn more about breastfeeding at school (intentions: M = 32.64, SD = 5.386/attitudes: M = 119.98, SD = 13.049). It is interesting that there is a positive correlation (r = 0.203, *p* < 0.05) between the desire to learn more about breastfeeding at school and the place of residence, meaning that students from larger cities are less likely to have the desire to learn more about breastfeeding at school when compared to students from rural areas. Formula advertising messages and promotions have reached vulnerable, economically disadvantaged sectors [42] Some researchers argue that in populations of low income women, where everyday life is full of danger and fear, it is understandable that breastfeeding is not considered practical [43]. Most research has noted a rural disadvantage in breastfeeding initiation, which can be explained by lower economic resources, work environments, and social support among rural minority postpartum women, related to mothers’ ethnicity status [44]. It is apparent that the mother’s place of residence directly affects the socioeconomic, psychological and cultural conditions in which she lives, and which then affect the decision to breastfeed. In that context, many studies confirm the implication of cultural and traditional practices on lactation and breastfeeding [45]. Our results, that show that students from rural areas who want to learn more about breastfeeding at school have more positive intentions and attitudes toward breastfeeding, are related to traditional culture of the Croatian village which is oriented to the natural nutrition of a child who is in an urban area subject to the influence of negative propaganda, not only that of the pharmaceutical industry, but also of modern-day narcissistic culture and body eroticism [46].

Another variable that showed an effect on breastfeeding behavior in previous research is longevity of being breastfed in childhood. The authors found that women who were breastfed as infants were more likely to breastfeed their children than women who were not breastfed in their early childhood [47]. It is well known that intentions are strongest predictors of future behavior, which would allow us to expect that our students who were breastfed as infants have more positive intentions towards breastfeeding than students who were not breastfed or who were breastfed for a shorter time. Results showed that female students who were not breastfed or who were breastfed up to 3 months of age have less positive intentions (M = 30, SD = 6.759) and attitudes (M = 120.82, SD = 14.063) towards breastfeeding compared to students who were breastfed for more than 3 months (intentions: M = 34.66, SD = 5.286/attitudes: 129.71, SD = 12.872).

To examine concurrent validity, the Pearson correlation coefficient was calculated. Hypotheses were that the breastfeeding intention scale and breastfeeding attitude scale are moderately to highly correlated and that the knowledge test would also have a positively moderate to high correlation with attitude and intention scales. Pearson’s correlation coefficient revealed a moderate correlation between intentions and attitudes which indicates that students who have more positive attitudes towards breastfeeding also have more positive intentions to breastfeed. Also, there was a moderate significant correlation between the knowledge test and attitude scale. The correlation between knowledge and intentions was low but significant. It is not unusual that knowledge does not have as strong a correlation with intentions as does attitude. It often appears that one does know all the benefits of breastfeeding and declare himself as an approver of, e.g., breastfeeding in public, but when asked if he would have breastfeed/support breastfeeding in public himself, then the answer is often the opposite.

In conclusion, the authors could not find a breastfeeding intention, attitude and knowledge questionnaire that could be applied for evaluation of educational activities in schools; therefore, they started to develop their own questionnaire. The validated breastfeeding questionnaire for young people allows for an objective monitoring of the effectiveness of education, but also the comparison of the efficiency of different educational models as well as a comparison of results from different backgrounds, which should speed up the process of developing a unique breastfeeding education plan and its implementation in the educational plan and program of primary and secondary school students [48]. The questionnaire is free of charge and available upon request to the authors of the study, with an explanation of the use intention.

Limitations: Since the subjects of the study were secondary-school students it was not possible to examine the predictive validity of BIAKQ. It would be interesting to test whether breastfeeding intentions, attitudes and knowledge of adolescents can predict their choice of breastfeeding method, longevity of breastfeeding, etc., once they face the experience of being a parent. For future research, it is also recommended to examine measurement characteristics of this questionnaire in different types of school and to calculate test-retest indicators.

## 8. Conclusions

The authors have developed a questionnaire to measure breastfeeding intentions, attitudes and knowledge among secondary-school students. This questionnaire is a reliable and valid measure to examine breastfeeding attitudes, intentions and knowledge among adolescents and could be very useful tool in examining differences between different types of schools and in the evaluation of breastfeeding education modules. Questionnaire should facilitate and accelerate the development of a unique and structured breastfeeding education program for both upper grade elementary and secondary-school students.

## Figures and Tables

**Table 1:** Demographic Characteristics and Experiences of Study Participants.

Participants´ characteristics	3rd Grade High School Students			4th Grade High School Students			4th Grade Medical Secondary School Students		
	M ± SD	*n*	%	M ± SD	*n*	%	M ± SD	*n*	%
Gender									
Male		44	60.7		45	40.5		13	24.1
Female		68	39.3		66	59.5		41	75.9
School achievement in previous school year									
Excellent		51	45.5		55	49.5		7	13
Very good		50	44.6		48	43.2		36	66.7
Good		10	8.9		7	6.3		7	13.0
Insufficient		1	0.9		1	0.9		0	0
Were you breastfed as a baby?									
Yes		99	88.4		97	87.4		47	87
No		7	6.3		6	5.4		7	13
Motivation to learn more about BF at school									
Yes		55	49.1		59	53.2		30	55.6
No		57	50.9		52	46.8		24	44.4
Age	16.71 ± 0.576			17.73 ± 0.466			17.24 ± 0.473		
16–17		107	95.5		31	27.9		42	77.8
18–19		5	4.5		80	72.1		12	22.2
Place of residence									
City		49	43.8		37	33.3		3	5.6
Small town		17	15.2		24	21.6		15	27.8
Village		46	41.1		50	45		36	66.7
Education level-mother									
Elementary school		3	2.7		5	4.5		10	18.5
Secondary school		65	58		55	49.5		36	66.7
BA degree		7	6.3		17	15.3		7	13.3
MA degree		34	30.4		30	27		1	1.9
Doctorate		3	2.7		4	3.6		0	0
Education level-father									
Elementary school		4	3.6		2	1.8		5	9.3
Secondary school		66	58.69		55	49.5		39	72.2
BA degree		3	9.8		22	19.8		7	13.0
MA degree		29	25.9		28	25.2		3	5.6
Doctorate		2	1.8		4	3.6		0	0
Longevity of being breastfed									
I don´t know/I was not breastfed		27	24.1		32	28.8		17	31.5
Up to one month of age		9	8		7	6.3		11	20.4
Up to three months of age		15	13.4		15	13.5		9	16.4
Up to six months of age		28	25		15	13.5		4	7.4
Up to one year of age		28	25		36	32.4		11	20.5
Up to 2 years of age		3	2.7		5	4.5		1	1.9
Longer than 2 years of age		2	1.8		1	0.9		1	1.9
Reason for discontinuation									
Mother believed I no longer need breastfeeding		29	25.9		36	32.4		2	3.8
Mother does not remember		23	20.5		21	18.9		20	37
I lost interest in breastfeeding		22	19.6		23	20.7		6	11.1
Other reasons		15	13.4		10	9.0		4	7.3
Less milk or “weak” milk		14	12.5		13	11.7		18	33.3
Mother started going to work		5	4.5		4	3.6		1	1.9
Issues with breastfeeding-pain due to biting a breast		4	3.6		4	3.6		3	5.6
Being around someone who breastfeeds									
Yes		55	49.1		36	32.4		52	96.3
No		57	50.9		75	67.6		2	3.7
Sources of information about BF ranked as the most important ones									
School		27	24.1		19	17.1		13	24.1
Mother		26	23.2		19	17.1		7	13
Internet		13	11.6		19	17.1		7	13
Magazines		18	16.1		22	19.8		10	18.5
Friends		18	16.1		31	27.9		12	22.2
TV		10	8.9		1	0.9		5	9.2

**Table 2:** Number of extracted components with Eigenvalues above 1 regarding cumulative and total variance for breastfeeding intention scale.

Number of Extracted Components	Eigenvalue	Proportion of Total Explained Variance	Cummulative Explained Variance
1	2.978	29.78	29.78
2	1.625	16.247	46.027
3	1.169	11.693	57.719
4	1.054	10.541	68.26

**Table 3:** Factor loadings and items distributions after Promax rotation.

Items	Factors			
	F1	F2	F3	F4
Returning to work would not make me stop breastfeeding.	0.858	0.321	0.179	0.181
I would stop breastfeeding my child as soon as I started to work, even if the child still desires	0.753	0.087	−0.011	0.56
If my partner would have helped me and brought child to my workplace, I would breastfeed	0.674	0.084	0.351	0.048
I intend to breastfeed after the call, i.e., according to the child’s request	0.175	0.74	0.089	−0.076
I would not breastfeed my child after he/she turns two, even if the child so desires	0.027	0.705	−0.183	0.351
I would continue with breastfeeding after my child turns one if the child so desires.	0.496	0.694	0.121	0.478
I would not breastfeed in public, for example, in a restaurant or in a park.	0.151	−0.051	0.857	0.208
Regardless of where I am, (home, park, facility) if my child demands breastfeeding, I would breastfeed	0.392	0.158	0.851	0.139
After the delivery, I would not try to establish breastfeeding. I would bottle-feed my child with formula milk.	0.129	0.035	0.281	0.779
After returning to work, I would instantly started to bottle-feed the baby with formula milk	0.348	0.483	−0.039	0.721

**Table 4:** Arithmetic mean and variance if item deleted, corrected item total-correlation and Cronbach α if item deleted.

Items	M	V	Corrected Item-Total Correlation	Cronbach’s Alpha If Item Deleted
1. After the delivery, I would not try to establish breastfeeding. I would bottle-feed my child with formula milk.	28.48	30.702	0.321	0.699
2. I would stop breastfeeding my child as soon as I started to work, even if the child still desires milk.	29.28	28.941	0.462	0.678
3. I would not breastfeed in public, for example, in a restaurant or in a park, even if my child so desires.	29.19	29.379	0.278	0.709
4. After returning to work, I would instantly start to bottle-feed the baby with formula milk.	29.72	28.941	0.438	0.681
5. I would continue with breastfeeding after my child turns one if the child so desires.	30.04	25.855	0.569	0.652
6. Regardless of where I am (home, park, facility), if my child demands breastfeeding, I would breastfeed.	29.11	28.511	0.448	0.678
7. Returning to work would not make me stop breastfeeding.	29.27	28.72	0.491	0.674
8. I would not breastfeed my child after he/she turns two, even if the child so desires.	30.68	30.22	0.214	0.72
9. If my partner would have helped me and brought child to my workplace, I would breastfeed.	29.9	28.792	0.35	0.695
10. I intend to breastfeed after the call, i.e., according to the child’s request.	29.41	30.965	0.219	0.715

**Table 5:** Comparison of intentions, attitudes and knowledge total scores between non-educated 3rd-grade high school students and educated 4th-grade high school students and between educated 4th-grade high school students and medical secondary school students.

		**Intentions**				
**Group**	***n***	**M**	**SD**	**t**	**df**	***p***
3rd-grade high school students	112	32.79	5.82	3.132	221	0.005
4th-grade high school students	111	35.24	5.901			
4th-grade medical secondary school students	54	25.18	2.65	2.39	163	0.05
4th-grade high school students	111	28.6	4.11			
		**Attitudes**				
**Group**	***n***	**M**	**SD**	**t**	**df**	***p***
3rd-grade high school students	112	122.31	13.957	−3.963	221	0.001
4th-grade high school students	111	129.74	14.030			
		**Knowledge**				
**Group**	***n***	**Mean Rank**	**U**	***p***		
3rd-grade high school students	112	94.26	8.203	0.01		
4th-grade high school students	111	128.80				
4th-grade medical secondary school students	54	53.95	1.4285	0.01		
4th-grade high school students	111	90.86				

**Table 6:** Factor loadings and item distributions after Varimax rotation and associated Cronbach alfa coefficient.

Items	Components						
	F1	F2	F3	F4	F5	F6	F7
8. Breastfeeding in public should be prohibited.	0.829	0.203	0.044	0	0.054	0.159	0.025
14. Breastfeeding in public is natural.	0.785	0.061	0.066	0.17	0.089	−0.025	−0.014
12. Women should not breastfeed in public.	0.739	0.137	0.059	0.092	0.035	0.155	−0.17
23. People who have had the opportunity to see a woman who breastfeeds in the public are more willing to breastfeed in public themselves.	0.654	0.136	0.102	0.027	0.124	−0.039	0.019
29. Breastfeeding in public expands and promotes breastfeeding nutrition culture as the best food for a child.	0.627	0.421	0.032	−0.056	0.132	0.04	0.032
3. In my opinion it is socially acceptable that a mother breastfeeds her hungry child in Church.	0.541	−0.047	−0.058	0.381	−0.088	−0.009	0.142
35. Breastfeeding in public increases the tolerance and understanding of breastfeeding.	0.539	−0.003	0.04	0.192	−0.176	0.332	0.03
38. Breastfeeding in public is a part of breastfeeding promotion.	0.442	0.168	−0.204	0.3	−0.029	0.111	0.22
27. The duty of child’s father is to monitor the condition of his partner and make sure that she is eating and resting enough.	0.128	0.792	0.053	0.096	−0.003	−0.072	−0.098
24. One of the roles of a father in a child’s first year of life is to provide support and all the necessary help to the mother.	0.25	0.69	0.033	−0.004	0.111	0.193	−0.06
20. The father does not play an important role in the child’s life while the child breastfeeds.	0.147	0.555	0.155	0.06	−0.073	0.394	0.056
16. Awareness of breastfeeding could be of great help to a father in helping a mother who breastfeeds.	0.361	0.537	−0.059	0.367	−0.18	0.102	0.037
11. A child’s father should definitely use a part of the maternity allowance to help the mother with breastfeeding and childcare.	−0.043	0.485	0.221	0.453	0.249	−0.122	0.031
6. Fathers attending breastfeeding support groups can learn how to help the mother in starting and maintaining breastfeeding.	0.278	0.451	0.043	0.359	−0.151	−0.013	0.037
34. Only women need to learn about breastfeeding and the impact of breastfeeding on child development since a child is a woman’s responsibility	−0.021	0.426	0.225	0.329	−0.196	0.17	0.1
18. On the day of delivery, the mother should not breastfeed because she needs to rest.	0.088	−0.004	0.838	−0.017	0.111	0.247	0.151
25. The mother should first breastfeed the baby the day after delivery.	−0.072	0.187	0.791	−0.005	0.079	−0.042	−0.102
36. The mother must not breastfeed the child on a day of birth.	0.122	0.11	0.754	0.038	−0.091	0.274	0.151
39. The mother should first breastfeed her baby within an hour of giving birth.	0.122	0.02	0.705	0.234	0.095	0.002	0.13
32. The law should prevent disruption of a mother who breastfeeds in a public.	0.057	0.145	0.011	0.676	0.128	0.229	0.01
2. I think that a father should help his partner who is breastfeeding and works and bring a baby to its mother during her breastfeeding break.	0.282	0.123	0.07	0.611	0.087	0.253	−0.087
19. I agree that it is ok if a woman breastfeeds at her workplace during the breastfeeding break.	0.473	0.041	0.198	0.568	−0.07	−0.012	0.185
13. The employer should provide a space where the employed mothers can breastfeed their child or use a breast pump without interruption, regardless of whether or not he or she is obliged to do so by law.	0.357	0.288	0.17	0.506	0.021	−0.289	0.162
26. Children fed with mother’s milk are healthier than babies fed with formula.	0.038	0.087	0.008	−0.064	0.817	0.001	0.096
31. Milk formula is not an adequate substitute for breast milk.	0.004	−0.026	0.096	0.202	0.738	−0.049	−0.079
10. A mother who formula feeds her baby misses a part of maternity enjoyment.	0.125	−0.099	0.054	−0.021	0.687	−0.046	−0.011
4. Breastfeeding negatively affects the mother’s working abilities and career.	0.205	0.148	0.091	0.151	−0.065	0.719	0.095
1. I agree with the attitude that it is beautiful to see a mother who breastfeeds.	−0.071	−0.044	0.203	0.102	−0.029	0.587	0.113
5. It is not profitable to invest in breastfeeding.	0.417	0.262	0.059	0.13	0.211	0.501	0.132
30. A man feels neglected by a spouse who breastfeeds.	0.212	0.285	0.027	−0.284	−0.253	0.422	−0.088
15. It is justified to breastfeed a child older than 2 years of age.	0.084	0.005	0.113	0.031	−0.061	−0.099	0.859
21. It is not good to breastfeed a baby for more than two years because it increases the attachment of the baby to the mother.	−0.035	−0.05	0.097	−0.001	−0.091	0.133	0.729
9. It is wrong to breastfeed a child older than one year.	0.031	0.01	0.049	0.131	0.293	0.261	0.692
Cronbach alfa coefficient	0.843	0.772	0.82	0.716	0.693	0.576	0.7

**Table 7:** Comparison of total scores of intentions and attitudes of students who want to learn more about breastfeeding at school and students who do not want to learn more about BF at school (*n* = 223) and between female students who were not breastfed or who were breastfed up to 3 months and students who were breastfed for longer than 3 months (*n* = 68).

		**Intentions**				
**Group**	***n***	**M**	**SD**	**t**	**df**	***p***
Students who want to learn more about BF at school	114	35.32	6.235	−3.42	221	0.05
Students who do not want to learn more about BF at school	109	32.64	5.386			
Female students who were breastfed for more than 3 months	35	34.66	5.286	−3.175	66	0.05
Female students who were not breastfed or who were breastfed up to 3 months	33	30	6.759			
		**Attitudes**				
**Group**	***n***	**M**	**SD**	**t**	**df**	***p***
Students who want to learn more about BF at school	114	131.77	13.374	−6.659	221	0.01
Students who do not want to learn more about BF at school	109	119.98	13.049			
Female students who were breastfed for more than 3 months	35	129.71	12.872	−2.723	66	0.05
Female students who were not breastfed or who were breastfed up to 3 months	33	120.82	14.063			
